# Analysis of the sacroiliac joint vacuum phenomenon in adolescent thoracic idiopathic scoliosis (Lenke types 1 and 2)

**DOI:** 10.1097/MD.0000000000034487

**Published:** 2023-08-25

**Authors:** Tadatsugu Morimoto, Yusuke Yamamoto, Satoshi Suzuki, Mitsuru Yagi, Takaomi Kobayashi, Masaaki Mawatari, Morio Matsumoto, Kota Watanabe

**Affiliations:** a Department of Orthopaedic Surgery, Faculty of Medicine, Saga University, Saga, Japan; b Department of Orthopaedic Surgery, Faculty of Medicine, Nara Medical University, Nara, Japan; c Department of Orthopaedic Surgery, Keio University School of Medicine, Tokyo, Japan.

**Keywords:** adolescent idiopathic scoliosis, joint hypermobility, sacroiliac joint, sacroiliac joint degeneration, vacuum phenomenon

## Abstract

The sacroiliac joint (SIJ) is the largest axial joint in the human body, and the SIJ vacuum phenomenon (SIJ VP) is a common finding in computed tomography studies of the abdomen, pelvis, and lumbosacral spine in adults, with the incidence increasing with age. Adolescent idiopathic scoliosis (AIS) is an abnormal spinal curvature that appears during adolescence and places abnormal stress on the SIJs. This retrospective observational study aimed to investigate the incidence of the SIJ VP in thoracic AIS (Lenke types 1 and 2). Sixty-seven patients with AIS (age: 12–19 years) and 76 controls (age: 11–19 years) were retrospectively analyzed to investigate SIJ VP, subchondral bone cysts, and SIJ degeneration (Eno classification: type 0, no degenerative change; type 1, mild degenerative changes; type 2, substantial degenerative changes; and type 3, ankylosis). SIJ degeneration was defined as type ≥ 2. The association between SIJ VP, cysts, SIJ degeneration, and sagittal/coronal spinopelvic alignment was assessed. SIJ VP (59% vs. 35.5%, *P* < .01), cysts (32.8% vs. 1.3%, *P* < .01), and SIJ degeneration (3.2% vs. 2.6%, *P* = .823) differed significantly between the 2 groups. There were 0 cases of SIJ ankylosis (Eno classification type 3) in both groups. The VP was not correlated with lumbar lordosis, sacral slope, or Cobb angle. All lumbar modifier type C belonged to the VP present group, whereas none to VP absent group. Our results suggest an association between AIS and SIJ VP and SIJ cysts. SIJ VP and SIJ cysts in AIS may be caused and accelerated by abnormal mechanical stress on SIJ due to spinal deformity.

## 1. Introduction

The sacroiliac joint (SIJ) is the largest axial joint in the human body that transmits gravitational load from the spine to the pelvis and lower limbs.^[1,2]^ The SIJ vacuum phenomenon (SIJ VP) is a common finding in computed tomography (CT) studies of the abdomen, pelvis, and lumbosacral spine in adults, with the incidence increasing with age.^[3,4]^

VP is influenced by the chemical properties of nitrogen gas, the biological properties of synovial joints, and the laws (Henry’s and Boyle’s laws) governing the pressure, volume, and solubility of gases in an enclosed space.^[5,6]^ VP can be induced in joints by trauma, infection, osteonecrosis, calcium pyrophosphate deposition disease, distraction in positioning, and generalized joint hypermobility, which alter the joint pressure or volume.^[3,7–10]^ The incidence of VP in middle-aged and elderly individuals ranges from 35% to 69%,^[9]^ most likely due to age-related morphological change, as well as obesity, pregnancy, and childbirth, affecting these degenerative changes in SIJ.^[7–9]^

In contrast, the incidence of SIJ VP among young females was reported as 14% (<39 years) by Faflia et al,^[9]^ 33% (<40 years) by Lo et al,^[8]^ and 30% (10–20 years) by You et al^[11]^ Adolescent idiopathic scoliosis (AIS) is an abnormal spinal curvature that appears during adolescence, which places abnormal stress on the SIJs and may result in a higher incidence of SIJ VP than that in healthy individuals in the same age group.

In AIS, the Lenke classification system commonly used in pathological assessment and treatment decision-making includes 6 curve types, a lumbar spine modifier, and a sagittal thoracic modifier.^[12]^

**Figure 1.**
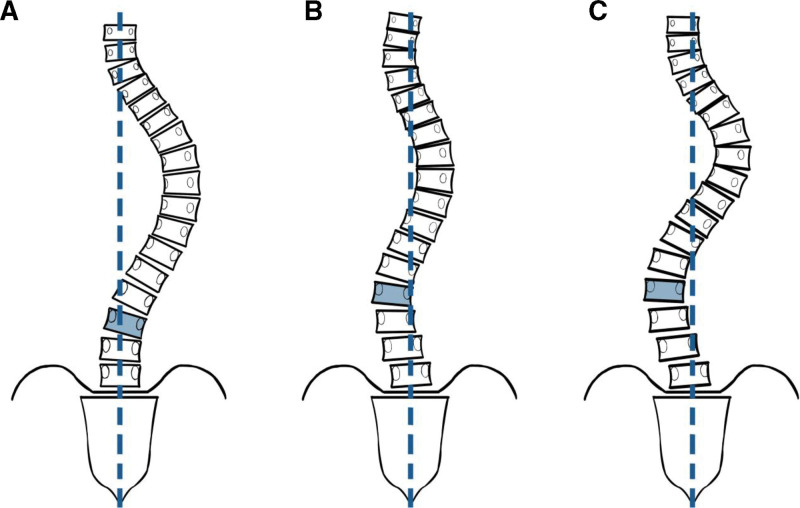
Lenke classification: lumbar modifiers (types A, B, and C). Type A: CSVL passes between the pedicles of the apical lumbar vertebrae. Type B: CSVL makes contact with the apical vertebral body. Type C: CSVL is completely medial to the vertebral body. CSVL = central sacral vertical line.

The lumbar spine modifier (A, B, or C) was evaluated according to the Lenke classification to grossly quantify the severity of the lumbar curve, which describes the relationship between the central sacral vertical line (CSVL) and the apical lumbar vertebra (type A, CSVL passes between the pedicles of the apical lumbar vertebrae; type B, CSVL makes contact with the apical vertebral body; and type C, CSVL is completely medial to the vertebral body) (Fig. 1). Thus, the apical lumbar vertebrae are located most laterally in type C, which may cause greater stress on the SIJ. This study aimed to investigate the incidence of VP and related factors in AIS.

## 2. Methods and patients

Our Institutional Review Board (reference #20090042) approved the review of patient data for this retrospective observational study. Informed consent was obtained in the form of opt-out on the website.

### 2.1. Patients

#### 2.1.1. AIS group.

Inclusion criteria were consecutive female patients with AIS (Lenke types 1 and 2: main curve in the thoracic vertebrae; side: right; all cases convex) who underwent posterior correction and fusion between January 2012 and September 2014, and those who underwent preoperative spinopelvic CT imaging. Male patients were excluded from the study because most of the AIS surgery cases were female.

#### 2.1.2. Control group.

Inclusion criteria were consecutive female patients treated in the emergency department from January to December 2018 and cases of abdominopelvic CT imaging for various indications.

Exclusion criteria were male patients for comparison with the AIS group, severe scoliosis, after spinal surgery, and poor image quality.

Finally, the data from 61 patients with AIS (average age: 15 years; range: 12–19 years) and 76 controls (average age: 15 years; range: 11–19 years) were analyzed.

Clinical data and imaging findings of the applicable patients were retrospectively reviewed.

### 2.2. Image acquisition

Abdominopelvic CT images were obtained using a 64-slice detector CT scanner (Canon Aquilion; Canon Medical System Co., Tochigi, Japan) as 1.3 mm-thick axial slices and reviewed in the bone window setting (window: 2000; level: 200). The acquisition parameters were as follows: tube voltage of 120 kVp, automatic exposure control at standard deviation value of 15, which is routinely used in clinical practice. The CT dose index volume and dose length product of the CT scans were recorded for each patient. The mean CT dose index volume and dose length product (±SD) were 5.1 ± 1.3 mGy and 310.8 ± 89.3 mGy.cm, respectively.

### 2.3. CT evaluation of VP, subchondral bone cysts, and SIJ degeneration according to the Eno classification

**Figure 2.**
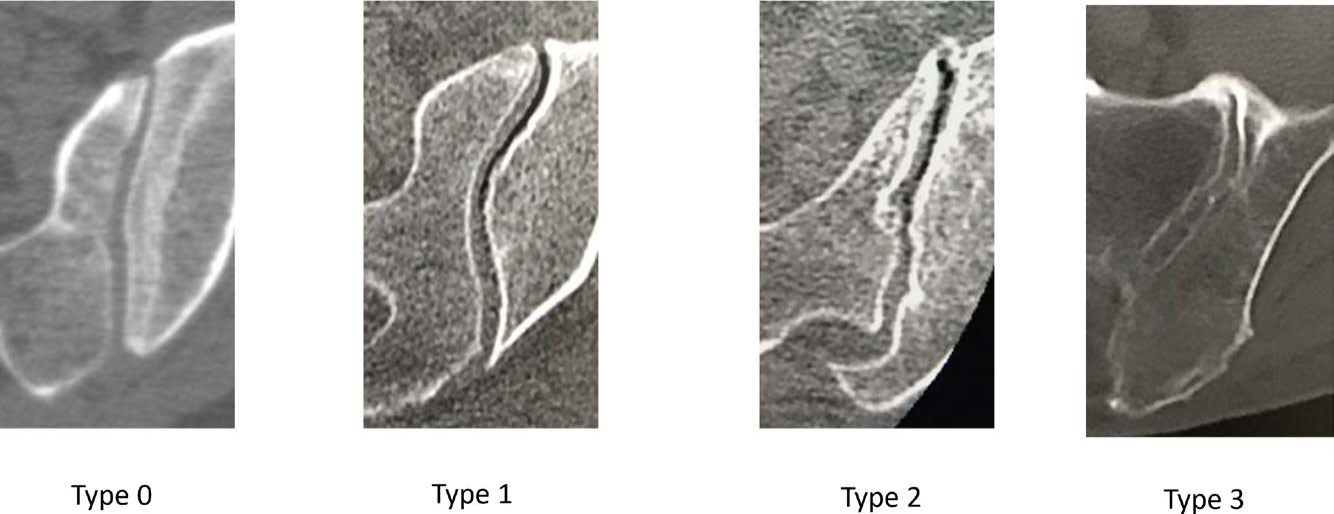
Sacroiliac joint degeneration according to Eno classification. (A) Type 0: no evidence of degenerative changes. (B) Type 1: mild degenerative changes (mild subchondral sclerosis, minimal osteophyte formation, and subtle joint space narrowing). (C) Type 2: substantial degenerative changes without ankylosis (large bridging osteophyte formation). (D) Type 3: sacroiliac joint ankylosis.

Axial CT imaging was performed to evaluate VP, subchondral bone cysts, and SIJ degeneration according to the Eno classification^[13]^ (Fig. 2) (type 0, no evidence of degenerative changes; type 1, mild degenerative changes with minimal osteophyte formation, mild subchondral sclerosis, and/or subtle joint space narrowing; type 2, substantial degenerative changes, including large osteophytes, substantial subchondral sclerosis, and/or definite joint space narrowing without ankylosis; and type 3, SIJ ankylosis). We defined type 2 or higher as “with SIJ degeneration.” VP and cysts were also evaluated as findings of SIJ degeneration that were not included in the Eno classification.

### 2.4. Sagittal and coronal spinal alignment

Lumbar lordosis (LL), sacral slope (SS), and the Cobb angle were measured in the whole spine of patients with AIS preoperatively in the standing position. The mean effective dose for spine radiographs in the sagittal and coronal planes is 1.96 (0.46–3.43) mGy and 0.92 (0.44–2.14) mGy, respectively.^[14]^ The coronal plane of the AIS was evaluated using the Lenke Classification of Lumbar Modifiers (A, B, and C).^[12]^

### 2.5. Statistical analysis

For comparison of qualitative data, the Fisher’s exact test or chi-square test was used, with description statistics expressed as “(number) percentage.” For comparison of quantitative data, the Mann–Whitney *U* test or Student’s *t* test was used, with description statistics expressed as “the median (interquartile range)” or “the mean ± SD,” respectively. SPSS statistics v. 27 and Microsoft Excel were used for the statistical analyses of the data. Statistical significance was set at *P* < .05.

## 3. Results

### 3.1. VP, cysts, and SIJ degeneration according to Eno classification

VP was observed in 59% (36/61) and 35.5% (27/76) of patients in the AIS and control groups, respectively (*P* < .01). Lenke types 1 and 2 showed VP in 62.2% (28/45) and 68.8% (11/16), respectively, with no significant difference (*P* = .64). Cysts were observed in 32.8% and 1.3% of patients in the AIS and control groups, respectively (*P* < .001) (Table 1). SIJ degeneration (Eno classification type 2≥) was observed in 3.2% (2/61) and 2.6% (2/76) of the patients in the AIS and control groups, respectively, with no significant difference (*P* = .823) (Fig. 1). There were 0 cases of SIJ ankylosis (Eno classification type 3) in both groups.

**Table 1:** Comparison of clinical characteristics, Eno classification, VP, cysts, and SIJ degeneration between the AIS and control groups.

	AIS (n = 61)	Control (n = 76)	*P* value
Age, yr	15.0 (13.0–16.0)	17.0 (14.0–18.0)	.010‡
Female, n (%)	61 (100)	76 (100)	NA
Eno classification, n (%)			
Type 0	32 (52.5)	46 (60.5)	.638†
Type 1	27 (44.3)	28 (36.8)	
Type 2	2 (3.2)	2 (2.6)	
Type 3	0	0	
VP, n (%)	36 (59.0)	27 (35.5)	.006†
Cysts, n (%)	20 (32.8)	1 (1.3)	<.001*
SIJ degeneration (≥ type 2), n (%)	2 (3.2)	2 (2.6)	.823*

AIS = adolescent idiopathic scoliosis, Cysts = subchondral bone cysts, LL = Lumbar lordosis, NA = not-available, SIJ degeneration = substantial degenerative changes, including large osteophytes, substantial subchondral sclerosis, and/or definite joint space narrowing without ankylosis, SS = sacral slope, VP = vacuum phenomenon.

* Shown as number (percentage) and compared by using the Fisher’s exact test.

† Shown as number (percentage) and compared by using the chi-square test.

‡ Shown as the median (interquartile range) and compared by using the Mann–Whitney *U* test.

### 3.2. Association between VP and the spinopelvic parameters in AIS

No correlations were found between VP and LL, SS, or the Cobb angle (Table 2). All lumbar modifier type C (n = 7) belonged to the VP present group, whereas none to VP absent group (Table 2).

**Table 2:** Association between VP and spinal alignment in AIS patients (n = 61).

	VP present (n = 33)	VP absent (n = 28)	*P* value
LL, degree	53.2 ± 10.5	50.3 ± 11.7	.321†
SS, degree	39.7 ± 7.0	37.0 ± 7.5	.160†
Cobb angle, degree	54.2 ± 9.1	57.5 ± 12.4	.230†
Lumbar modifier, n (%)			
Type A	21 (63.6)	25 (89.3)	NA*
Type B	5 (15.2)	3 (10.7)	
Type C	7 (21.2)	0 (0)	

AIS = adolescent idiopathic scoliosis, LL = lumbar lordosis, NA = not-available, SS = sacral slope, VP = vacuum phenomenon.

* Shown as number (percentage) and compared by using the chi-square test.

† Shown as the mean ± standard deviation and compared by using the Student’s *t* test.

### 3.3. Relationship between the laterality of VP and the convex side in AIS

All cases had a right convex curve in the thoracic spine. The laterality of VP in AIS was concave, convex, and bilateral in four (11%), six (16%), and 27 (73%) patients, respectively. VP had no significant correlation with the convex side of scoliosis.

### 3.4. Representative cases

**Figure 3.**
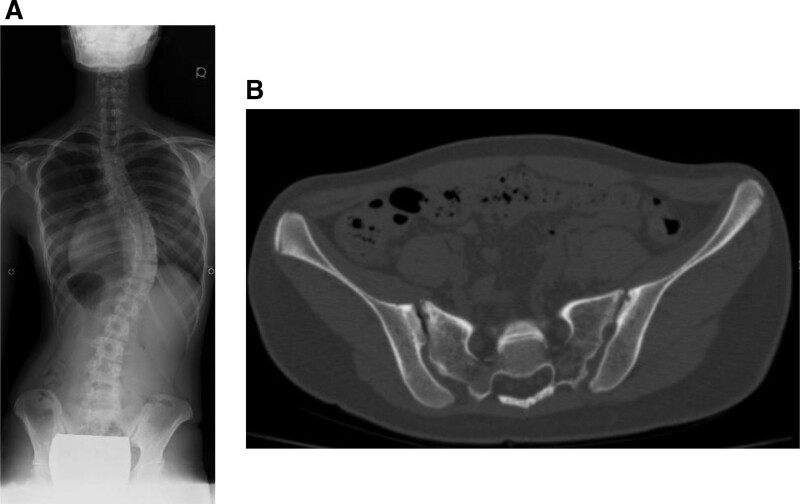
Representative case 1: 16-year-old female AIS patient. (A) Posterior anterior X-ray: Lenke classification type1, lumbar modifier type A, and Cobb angle of 56°. (B) Pelvic CT: sacroiliac joint degeneration Eno classification type 2, right side: VP, left side: Cyst (+) (white arrow). AIS = adolescent idiopathic scoliosis, CT = computed tomography, VP = vacuum phenomenon.

Case 1 is a 16-year-old girl with AIS (Lenke type 1, Lumbar modifier type A, and Cobb angle of 56°) (Fig. 3A). Pelvic CT showed Eno class type 2 SIJ degeneration, with VP on the right side and cyst on the left (Fig. 3B).

**Figure 4.**
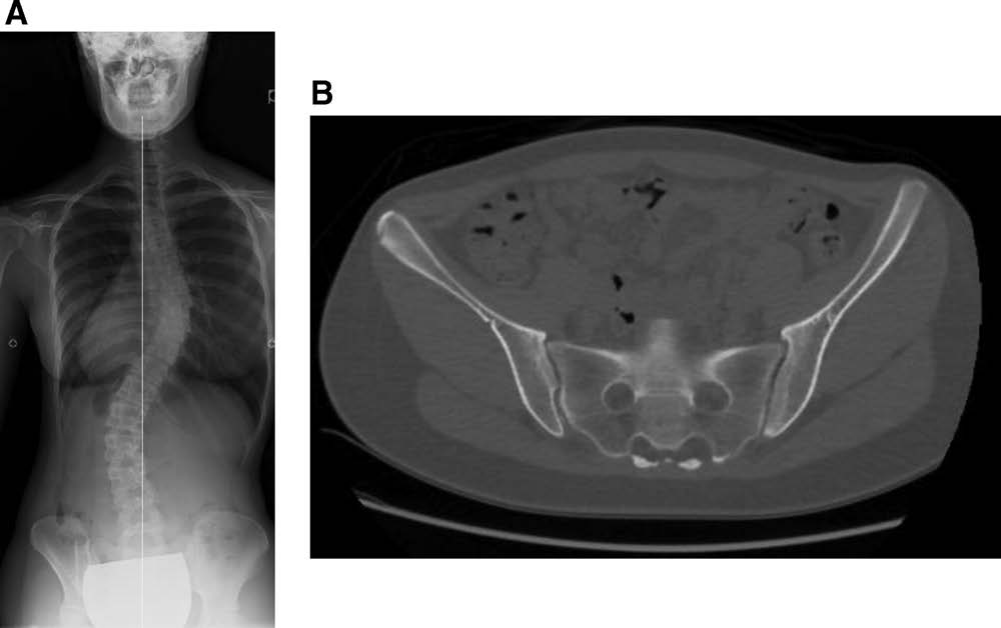
Representative case 2: 13-year-old female AIS patient. (A) Posterior anterior X-ray: Lenke classification type 1, lumbar modifier type C, and Cobb angle of 60°. (B) Pelvic CT: sacroiliac joint degeneration Eno classification type 1, both sides: VP. AIS = adolescent idiopathic scoliosis, CT = computed tomography, VP = vacuum phenomenon.

Case 2 is a 13-year-old girl with AIS (Lenke type 1, Lumbar modifier type C, and Cobb angle of 60°) (Fig. 4A). Pelvic CT showed Eno class type 1 SIJ degeneration with bilateral VP (Fig. 4B).

## 4. Discussion

To our knowledge, this is the first clinical study to investigate SIJ VP in female patients with AIS (main curve thoracic; Lenke types 1 and 2).

SIJ VP was relatively more common in patients with AIS (59%) than in the controls (35.5%). The incidence of SIJ VP among young females reportedly ranges from 14–33%.^[8,9,11]^ These rates are similar to those found in our study, suggesting that SIJ VP in young females may indicate early degeneration and physiological findings. The high prevalence of SIJ VP among young females with AIS may be due to abnormal loading of SIJ due to spinal deformity, anatomy of the SIJ, and joint hypermobility. Abnormal mechanical stress on SIJ due to scoliosis may also accelerate the occurrence of SIJ VP. It has been reported that the biomechanical stress on SIJ is increased in hip osteoarthritis due to the limited range of motion of the hip and leg length discrepancy, resulting in a higher incidence of VP and degeneration of SIJ.^[3,4]^ Lumbar and lumbosacral fusion also affect SIJ degeneration due to the mechanical stress on SIJ.^[15–17]^ Thus, biomechanical forces, such as shear stress on SIJ, can lead to the occurrence of SIJ VP in AIS, hip disorders, and in patients who undergo lumbar and lumbosacral fusion surgery. In addition, some studies have demonstrated that children with AIS have an asymmetrical gait,^[18–20]^ suggesting that abnormal stress on SIJ during gait may be a biomechanical factor that leads to the occurrence of SIJ VP in AIS.

From an anatomical standpoint, SIJ is oriented almost parallel to the plane of maximum loading.^[15]^ Therefore, the sacral bone is inclined in response to LL, which may be disadvantageous for bearing vertical load. Moreover, the female SIJ has a smaller joint surface and higher mobility, stress, and pelvic ligament strains than the male SIJ.^[16]^ These anatomical features of the female SIJ may be advantageous for childbirth. However, they may predispose females to degeneration, including VP. Increased joint mobility, which is relatively common in children and females, may be associated with joint volume changes when abnormal loading (e.g., traction) is applied to the joint. This may lead to VP. The finding that SIJ VP is not uncommon in adolescent females with AIS seems to support this theory. Generalised joint hypermobility, which is frequent in AIS,^[21,22]^ may also be a potential risk factor for SIJ VP.

SIJ cysts were more common in patients with AIS (32.8%) than in the controls (1.3%). It was hypothesized that cysts may be induced on osteoarthritic joint surfaces due to abnormal mechanical stress and subsequent microcracking, edema, and local bone resorption.^[23,24]^

The same mechanism may be associated with SIJ cysts in AIS, with increased mechanical stress on the SIJ derived from the cyst and AIS.

Regarding the association between VP and the spinopelvic parameters in AIS, there was no significant difference between the presence of SIJ VP and LL, SS, or the Cobb angle; however, all of lumbar modifier type C belonged to the VP present group, whereas none to the VP absent group (according to the Lenke classification).

Regarding the sagittal spinopelvic alignment, we speculated that greater LL and SS may increase the sacral inclination and SIJ degeneration; however, no significant correlation was observed. Such cases should be examined in the future. In contrast, Lenke type C showed significantly greater SIJ degeneration in the coronal alignment, suggesting that the mechanical factor of abnormal stress on SIJ can accelerate SIJ degeneration as the lumbar apical vertebrae are located most laterally in type C.

As for the relationship between the laterality of VP and the convex or concave side in AIS, since SIJ VP in AIS was mostly bilateral (73%), there was no significant relationship with the convex or concave side of scoliosis. Abnormal stress is exerted on SIJ in AIS, suggesting that there may be abnormal stress on the SIJ on both sides in AIS.

The present study had some limitations. First, the subjects were restricted to women since the reported sex ratio (male:female) of AIS with a Cobb angle of ≥30° requiring surgery was 1:10,^[25]^ and the overwhelming majority of AIS surgery cases were performed in women. Future studies should include male cases for more robust findings. Second, we did not assess clinical findings, such as lower back pain or general joint hypermobility, due to the retrospective study design. VP rarely induces symptoms, although patients may have early degenerative lesions and potential risk factors for future lower back pain. Cross-sectional and longitudinal studies are required to investigate the relationship between VP and lower back pain. Third, it is unclear whether SIJ VP in AIS is physiological, an early stage of degeneration; this must be investigated in future studies. Fourth, we did not assess pelvic incidence (PI), which is a key parameter in sagittal spinopelvic alignment, as PI could not be measured due to the gonadal protector placed on the pelvis. However, sacral inclination, which indicates an increased load on the SIJ, can be assessed satisfactorily in LL and SS. Fourth, SIJ anatomical variations have also been associated with SIJ degeneration and axial spondyloarthritis.^[26–28]^ In the present study, no SIJ anatomical variations in AIS were investigated, which is a subject for future research. Finally, the increase in abnormal stress on SIJ due to AIS has not been directly evaluated. It is necessary to evaluate this stress using the CT-based finite element method and other methods in the future.

In conclusion, the incidence of SIJ VP was significantly higher in patients with AIS. SIJ VP in AIS may be caused and accelerated by the anatomical features of SIJ (parallel to the loading direction of SIJ articular surface and sex-related morphology), joint mobility, and abnormal stress on SIJ due to spinal deformity in AIS. Further longitudinal studies are required to confirm the proposed mechanism of the association and clinical findings between AIS and VP or SIJ degeneration.

## Acknowledgements

We would like to thank Editage (www.editage.com) for English language editing.

## Author contributions

**Conceptualization:** Tadatsugu Morimoto.

**Data curation:** Tadatsugu Morimoto, Yusuke Yamamoto, Satoshi Suzuki, Takaomi Kobayashi.

**Formal analysis:** Tadatsugu Morimoto, Yusuke Yamamoto.

**Investigation:** Tadatsugu Morimoto, Takaomi Kobayashi.

**Methodology:** Tadatsugu Morimoto, Takaomi Kobayashi.

**Project administration:** Morio Matsumoto.

**Supervision:** Mitsuru Yagi, Masaaki Mawatari, Morio Matsumoto, Kota Watanabe.

**Validation:** Satoshi Suzuki.

**Writing – original draft:** Tadatsugu Morimoto.

**Writing – review & editing:** Masaaki Mawatari, Kota Watanabe.
